# The impact of methodology on the reproducibility and rigor of DNA methylation data

**DOI:** 10.1038/s41598-021-04346-w

**Published:** 2022-01-10

**Authors:** Detlev Boison, Susan A. Masino, Farah D. Lubin, Kai Guo, Theresa Lusardi, Richard Sanchez, David N. Ruskin, Joyce Ohm, Jonathan D. Geiger, Junguk Hur

**Affiliations:** 1 Department of Neurosurgery, Robert Wood Johnson Medical School, Rutgers University, Piscataway, NJ 08854 USA; 2 Department of Psychology and Neuroscience Program, Trinity College, Hartford, CT 06106 USA; 3 Department of Neurobiology, University of Alabama at Birmingham, Birmingham, AL 35294 USA; 4 Department of Biomedical Sciences, University of North Dakota School of Medicine and Health Sciences, Grand Forks, ND 58202 USA; 5 Knight Cancer Institute, Cancer Early Detection Advanced Research Center, Oregon Health and Science University, Portland, OR 97239 USA; 6 Dow Neurobiology Labs, Legacy Research Institute, Portland, OR 97232 USA; 7 Department of Genetics and Genomics, Roswell Park Comprehensive Cancer Center, Buffalo, NY 14263 USA; 8 Present Address: Department of Neurology, University of Michigan, Ann Arbor, MI 48109 USA; 9 Present Address: Division of Biological Sciences, Neurobiology Section, University of California San Diego, La Jolla, CA 92093 USA

**Keywords:** Methylation analysis, Neurology

## Abstract

Epigenetic modifications are crucial for normal development and implicated in disease pathogenesis. While epigenetics continues to be a burgeoning research area in neuroscience, unaddressed issues related to data reproducibility across laboratories remain. Separating meaningful experimental changes from background variability is a challenge in epigenomic studies. Here we show that seemingly minor experimental variations, even under normal baseline conditions, can have a significant impact on epigenome outcome measures and data interpretation. We examined genome-wide DNA methylation and gene expression profiles of hippocampal tissues from wild-type rats housed in three independent laboratories using nearly identical conditions. Reduced-representation bisulfite sequencing and RNA-seq respectively identified 3852 differentially methylated and 1075 differentially expressed genes between laboratories, even in the absence of experimental intervention. Difficult-to-match factors such as animal vendors and a subset of husbandry and tissue extraction procedures produced quantifiable variations between wild-type animals across the three laboratories. Our study demonstrates that seemingly minor experimental variations, even under normal baseline conditions, can have a significant impact on epigenome outcome measures and data interpretation. This is particularly meaningful for neurological studies in animal models, in which baseline parameters between experimental groups are difficult to control. To enhance scientific rigor, we conclude that strict adherence to protocols is necessary for the execution and interpretation of epigenetic studies and that protocol-sensitive epigenetic changes, amongst naive animals, may confound experimental results.

## Introduction

Epigenetic mechanisms, including alterations in DNA methylation, histone covalent post-translational modifications, and non-coding RNAs^1^, allow an organism to adapt to changes in environmental conditions. In particular, the epigenome of the central nervous system is responsive to dynamic changes in internal and external environments, thereby providing a foundation for processes as varied as memory formation or behavior, and when disrupted, it leads to the development of pathologies including epilepsy^2–4^. Indeed, epigenetic mechanisms such as DNA methylation have evolved to enable adaptive gene expression as well as contribute to the pathophysiology of disease initiation and progression. Significant and growing research efforts seek to identify key disease-associated epigenetic marks, based on the scientific premise that averting or reversing epigenetically driven pathological changes can prevent, diminish, or cure disease. Consequently, epigenetic therapeutic strategies are currently considered for clinical implementation in a wide variety of medical conditions^5,6^, and epigenetic therapies have already been implemented for the treatment of cancer^7–9^.

Detailed epigenome analyses applied to translational disease models typically find dozens to thousands of potential epigenetic modifications at gene regions, and the process of identifying causal factors is unavoidably challenging and time-consuming. Adding further to this complexity are observations of major differences in epigenetic signatures among different models of the same disease. For example, a recent study examined genome-wide DNA methylation levels without matching experimental protocols in three different animal models of epileptogenesis, each performed in a different laboratory, and found no meaningful common changes in DNA methylation associated across the three models, which led the authors to conclude that there was no mechanistic overlap among models^10^. However, one protocol-related contributing factor that has not been adequately considered in epigenetic studies is the comparison of differences between control and experimental tissue within a laboratory or between laboratories.

**Figure 1:**
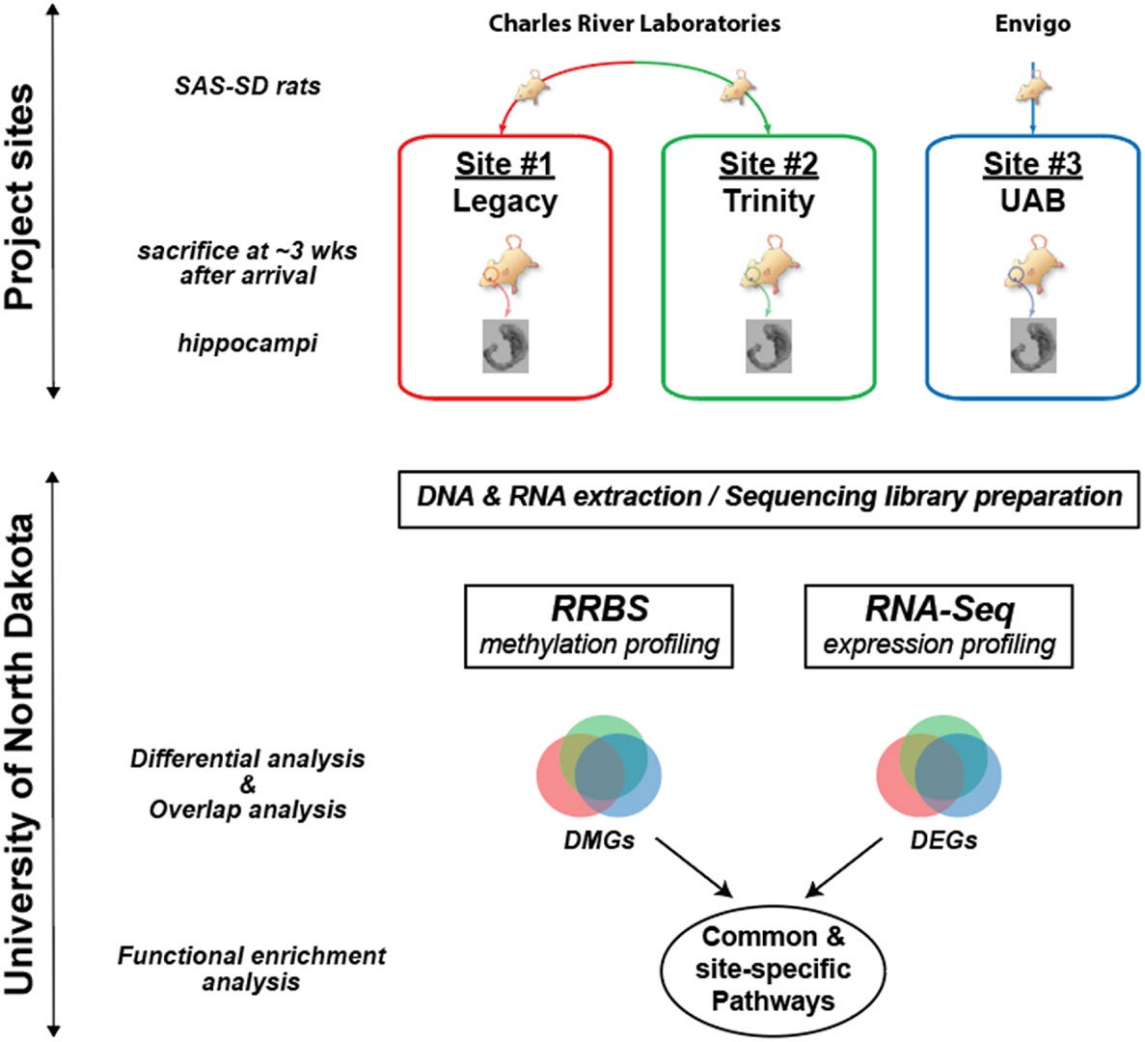
The overall workflow of the study. Animals were bred at three project sites: Site #1: Legacy Research, Site #2: Trinity College, and Site #3: the University of Alabama at Birmingham (UAB). Hippocampus was harvested from each animal and sent to the University of North Dakota for sequencing analysis. RRBS: reduced representation bisulfite sequencing. The workflow was created using Adobe Illustrator with the rat and hippocampus images obtained from http://en.wikimedia.org under the Creative Commons Attribution-ShareAlike 3.0 license.

To begin to address whether baseline experimental analysis of DNA methylation can be influenced by interlaboratory protocol-related confounds, we sought to compare DNA methylation marks in control wild-type tissue collected from three different laboratories. Hippocampal tissues were harvested and examined for DNA methylation and associated gene expression differences across the three laboratories (Fig. 1), minimizing protocol differences, and matching variables such as vendor, age, rat strain, and tissue processing method for analysis.

## Results

### Experimental and environmental factors

SAS-Sprague Dawley male rats were purchased from vendors (Charles River and Envigo) nearest to our three project sites; Legacy Research Institute in Portland, Oregon (Site #1), Trinity College in Hartford, Connecticut (Site #2), and the University of Alabama at Birmingham in Birmingham (UAB), Alabama (Site #3). We identified factors that are typically easy to match, factors that may not always be considered, and factors that are challenging to match (Table 1). A total of 28 factors were collected in four major areas including “before-each-laboratory”, “at-each-laboratory”, “up-to-sacrifice”, and “dissection”. Three factors (10.7%; distance from breeding site, caging shape and size, and chow vendor) were all unique at each site, while 25 (89.2%) factors were shared by two or more sites. Based on the number of matched variables, Site #1 and Site #2 were the most similar to each other, sharing 22 (78.6%) factors, while Site #3 shared the least numbers of factors with Site #1 (14; 50.0%) and Site #2 (15; 53.6%).

**Table 1:** Experimental and environmental factors.

	Factors	Site#1 (Legacy)	Site#2 (Trinity)	Site#3 (UAB)	Comparable sites		
					1	2	3
**Before each laboratory**	Animal vendor	Charles River	Charles River	Harlan (Envigo)	✔	✔	
	Vendor breeding site	Kingston, NY	Kingston, NY	Frederick, MD	✔	✔	
	Chow at breeding site	Purina LabDiet 5L79	Purina LabDiet 5L79	Teklad Global 18% Protein Rodent Diet	✔	✔	
	Distance from breeding site	4723 km	146 km	1173 km			
	Transit time	5 days in shipment (by truck) + 3 h time zone change	2 h 22 min	4 days (ordered placed 5–29–15, arrived 6–2–15) + 1 h time zone change	✔		✔
	Strain	SAS-SD (Sprague Dawley)	SAS-SD (Sprague Dawley)	SAS-SD (Sprague Dawley)	✔	✔	✔
	Sex	Male	Male	Male	✔	✔	✔
	Ordered at weight	226–250 g	226–250 g	226–250 g	✔	✔	✔
**At each laboratory**	Age at arrival	8.3 weeks	8.3 weeks	8 weeks	✔	✔	✔
	Single- or double-housed	Single	Single	Double	✔	✔	
	Caging: stand-alone cages or water & air piped in	Connected to individual ventilation	Stand-alone	Stand-alone		✔	✔
	Caging: shape and size	Rectangular 42.4Lx26.7Wx18.5D (cm)	Rectangular 26.9Lx21.6Wx14.2D (cm)	Rectangular 36.8Lx29.2Wx22.9D (cm)			
	Bedding type	Paper	Wood chip	Wood chip		✔	✔
	12 h:12 h light cycle	Yes	Yes	Yes	✔	✔	✔
	Chow	LabDiet 5001	LabDiet 5001	NIH open formula rat sterilizable diet	✔	✔	
	Chow vendor	Animal Specialties, Woodburn, OR	WF Fisher & Son, NJ	Teklad/Envigo, AL			
**Up to sacrifice**	Days from arrival to start handling	3 days	3 days	3 days	✔	✔	✔
	Days from arrival to sacrifice	18–19 days	18–19 days	27 days	✔	✔	
	Handling details	Gentle towel wrapping, stroking	Gentle towel wrapping, stroking	Gentle towel wrapping, stroking	✔	✔	✔
	Daily handling	Yes	Yes	Yes	✔	✔	✔
	Rats weighed day of sacrifice or earlier	One day prior	One day prior	On day of sacrifice	✔	✔	
	Sacrifice method	Rapid decapitation, no anesthesia	Rapid decapitation, no anesthesia	Rapid decapitation, no anesthesia	✔	✔	✔
**Dissection**	Whole hippocampus	Yes	Yes	Yes	✔	✔	✔
	Type of buffer	0.9% saline	0.9% saline	Artificial cerebrospinal fluid	✔	✔	
	Bubbled	No	No	Yes (95% O_2_ + 5% CO_2_)	✔	✔	
	pH checked or adjusted	No	No	No	✔	✔	✔
	How was buffer cooled	Refrigerated, then on ice	Refrigerated, then on ice	Refrigerated, then on ice	✔	✔	✔
	Was tissue weighed	No	No	No	✔	✔	✔

Site #1: Legacy Research, Site #2: Trinity College, and Site #3: the University of Alabama at Birmingham; SD: Sprague Dawley rat; ✔: comparable across sites. Animal handling was approved by the Institutional Animal Care and Use Committee (IACUC) at each of the three sites.

### Genome-wide profiling of DNA methylation and gene expression

Rats were 8.0 to 8.3 weeks of age at the time of arrival from the vendors. Entire hippocampi (from both hemispheres) were harvested from rats (n = 5–6) 18 to 27 days after arrival. The average body weights measured before animals were killed were 328.0 ± 15.9 g (n = 5), 310.8 ± 16.6 g (n = 6), and 337.9 ± 15.0 g (n = 6) for Sites #1, #2, and #3, respectively. Overall, these body weights were significantly different (ANOVA *p* value = 0.03), while only the pair of Site #2 and Site #3 was statistically significant in a pairwise comparison (Bonferroni corrected *p* value = 0.04). Whole hippocampi from each animal were surgically dissected and processed for deep sequencing.

**Figure 2:**
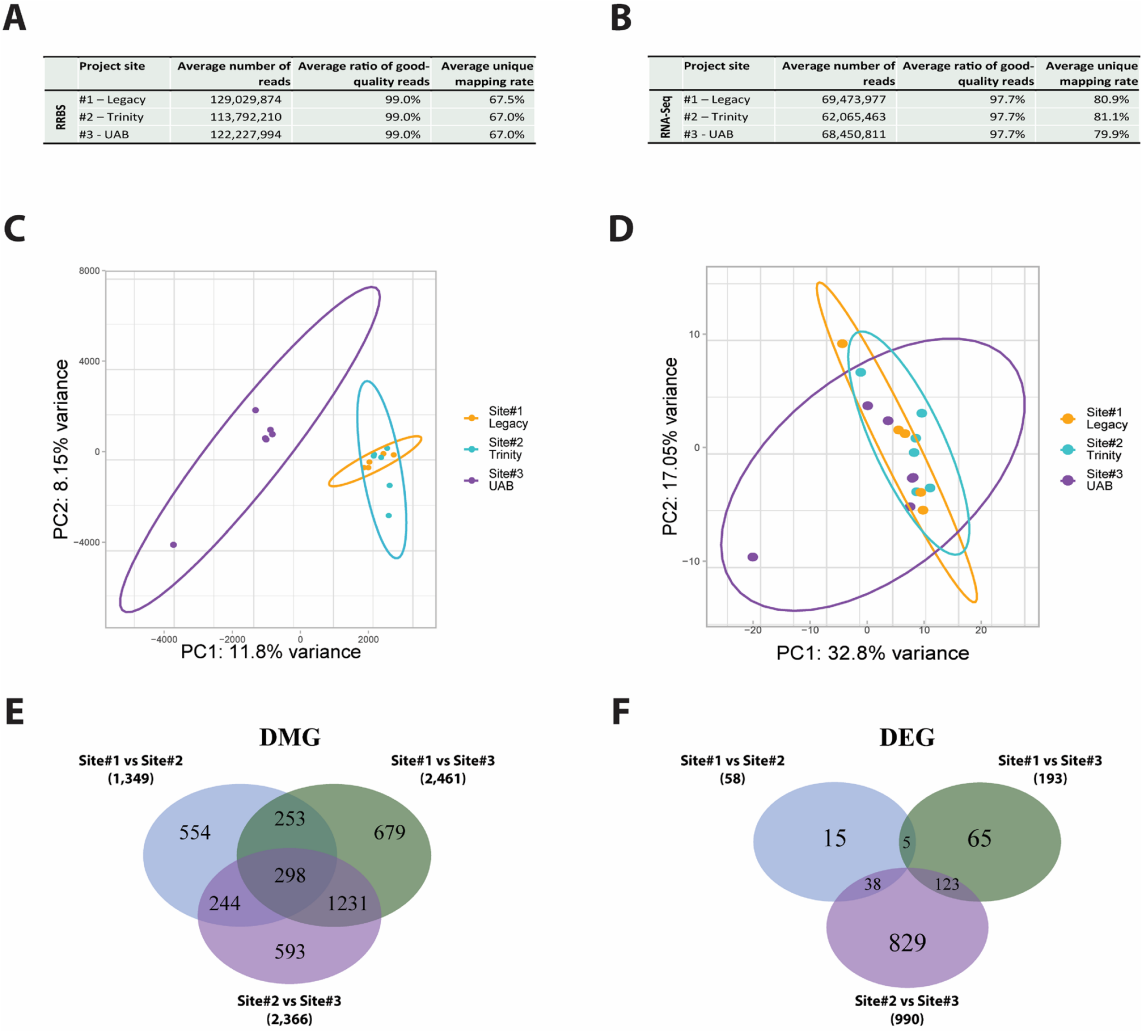
RRBS and RNA-Seq summary. The average number of sequencing reads, the average ratio of good quality reads, and the average unique mapping rates per sample are given for RRBS (**A**) and RNA-Seq (**B**). Principal component analysis (PCA) on RRBS (**C**) and RNA-Seq (**D**) was performed to examine the overall similarity among the samples. Differentially methylated genes (DMGs; [**E**]) and expressed genes (DEGs; [**F**]) were obtained between each pair of project sites and compared among the sets. Panel images were created using R (v4.0.3).

We obtained approximately 120 million 50-bp single-end reads per sample for DNA methylation profiling using reduced representation bisulfite sequencing (RRBS; Fig. 2A) and 66 million 50-bp paired-end reads per sample for gene expression profiling using RNA-Seq (Fig. 2B). The obtained sequencing reads were of high quality. A total of 8,779,630 CpG sites, corresponding to 38,185 genes, approximately 95% of 40,189 genes in the Rn6 rat genome annotation, were measured in at least one sample of the current RRBS dataset, and each sample included an average of 2,133,011 measured CpG sites (minimum 1,756,129 and maximum 2,646,863; Supplementary Table S1). A Principal Component Analysis (PCA) plot on the top 10% most variant CpGs (Fig. 2C) from the RRBS dataset illustrated that methylation profiles from Sites #1 and #2 were more similar, while samples from Site #3 were more divergent in terms of genome-wide methylation changes. Gene expression profiles (Fig. 2D) showed a higher congruence across all three sites.

### Hippocampal markers and cell-type abundance

**Figure 3:**
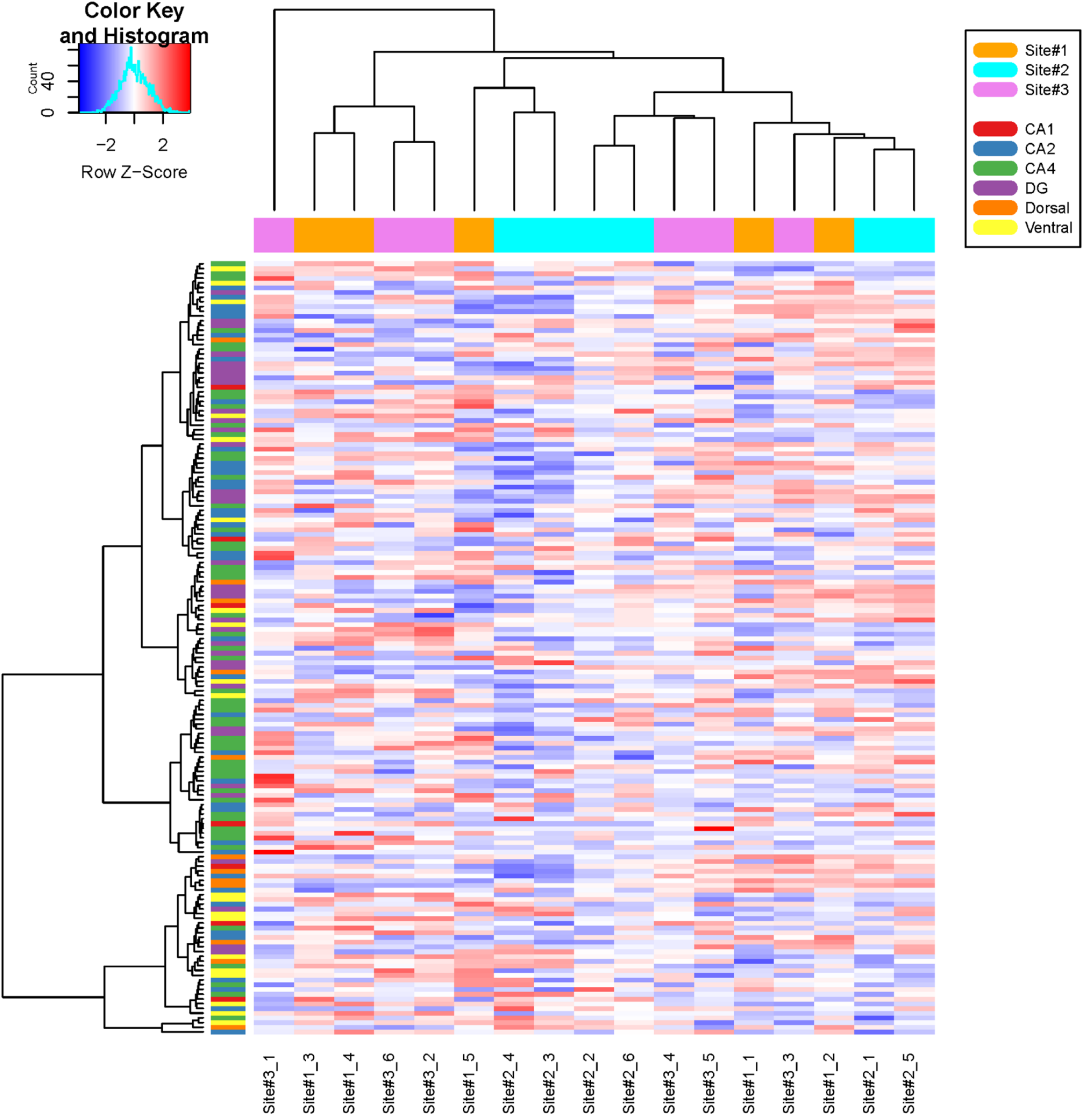
Expression heatmap of region-specific hippocampal markers. A heatmap of row-scaled 164 hippocampal markers was generated using Euclidean distance and complete linkage on the log_2_-transformed Fragments Per Kilobase Million (FPKM) data. Site #1: Legacy Research, Site #2: Trinity College, and Site #3: the University of Alabama at Birmingham. Hippocampal regions: CA1, CA2, CA4, DG (dentate gyrus), dorsal, and ventral regions. The image was created using R (v4.0.3).

As differences in DNA methylation and gene expression across sites were observed, we decided to examine the possibility that the differences originated from individual variance in surgical procedures; the result being possible differences in regions of the hippocampus being studied. We collected 184 region-specific gene expression markers (Supplementary Table S2), covering CA1, CA2, CA4, dentate gyrus, dorsal, and ventral regions in the hippocampus, from the Hipposeq database^11^ to examine the expression profiles in our dataset. Figure 3 illustrates the gene expression patterns of these marker genes, where no outstanding association between hippocampal regions was identified across samples. We also examined the average expression levels of known cell-type-specific neuronal marker genes, which were very comparable across three experimental sites (Supplementary Table S3). Cell-type abundance analysis using CIBERSORT^12^ on the expression data revealed that the majority of the cells were neurons, astrocytes, and oligodendrocytes as shown in Supplementary Fig. 1, and their compositions were not significantly different across the three project sites (Kruskal–Wallis *P* value > 0.05 for each cell type; Supplementary Table S4). These results suggest that there were no systematic variances in surgical procedures between experimental sites and collected cell-type compositions.

### Differential methylation and gene expression analysis

Both DNA methylation and gene expression data were analyzed in a pairwise fashion comparing samples from each site to both of the others. Differentially methylated CpGs (DMCs) were those with a methylation difference of > 25% and a false discovery rate adjusted *p* value < 0.01 by methylKit. Approximately 6.0% of DMCs were located in promoter regions, 34.6% in introns, 9.1% in exons, and 50.4% in intergenic regions (Supplementary Fig. 2). Each DMC was mapped to a gene with the shortest distance from its transcript starting site and differentially methylated genes (DMG) were identified as having at least one mapped DMC. We also examined the correlation between the methylation levels of DMCs and body weight before animals were killed to assess the effect of different body weight on methylation. While the majority of the Pearson correlations were not significant, a large portion of the DMCs between Site #1 and Site #3 showed a statistically significant correlation with body weight (Supplementary Fig. 3).

Differentially expressed genes (DEGs) were identified using DESeq2 and genes with a Benjamini–Hochberg adjusted *p* value < 0.05. Based on the total number of DMGs and DEGs, the comparison between Site #1 and Site #2 had the smallest numbers of DMGs (n = 1,49) and DEGs (n = 58), suggesting that these two sites had the most similar DNA methylation and gene expression profiles among the three sites, which is also supported by the hierarchical clustering of samples (Supplementary Fig. 4). The comparison between Site #2 and Site #3 was the most divergent with a total of 2366 DMG and 990 DEG identified. The comparison between Site #1 and Site #3 had a similar number of DMGs (n = 2461) but fewer DEGs (n = 193) than that of Site#1 vs Site#2. The complete lists of DMGs and DEGs are available in Supplementary Tables S5-S10 and their overlaps are illustrated in Venn diagrams (Fig. 2E,F). We also performed an analysis of differentially methylation regions (DMRs) in addition to DMGs using methylKit, which identified 24–77 DMRs between sites (Supplementary Tables S11-S13).

### Overlap between DEGs and DMGs

Genome-wide DNA methylation studies commonly report changes in DNA methylation in the absence of gene expression data. When combined with gene expression data, investigators often focus on the alterations in DNA methylation that can be inversely correlated with gene expression changes. To understand the relationship between epigenetic regulation and transcriptomic changes, we examined the overlap between DMGs and DEGs. Table 2 lists the overlaps at both gene and CpG site levels. The comparison between the two most similar sites (Site #1 Legacy and Site #2 Trinity) with the smallest numbers of DMGs (n = 1349) and DEGs (n = 58) includes no overlapping genes, while the other comparisons shared 1 to 5% of DMGs with DEGs. Even the comparison with the biggest overlap (Site #2 Trinity and Site #3 UAB) was not statistically significant (hypergeometric test, *p* value = 0.171). Approximately 53 to 59% of differential CpG sites showed an inverse relationship between the direction of methylation and expression changes such as increased methylation with down-regulated gene expression or decreased methylation with up-regulated gene expression. This is reflective of the nuances associated with the position of DNA methylation changes in relation to gene expression and strongly suggests that even in the minority of instances where DMG show a change in gene expression, the directionality of that gene expression change cannot and should not be inferred based on whether DNA methylation is increased or decreased at a particular site.

**Table 2:** Overlap between DMGs and DEGs.

Comparison	Gene level			CpG site level			
	DMGs	Overlap	DEGs	# CpG sites (Overlap)	Same direction	Opposite direction	Opposite direction (gene)
Site #1 vs Site #2	1349	0	58	0	0	0	0
Site #1 vs Site #3	2461	33	193	59	24	35	20
Site #2 vs Site #3	2366	127	990	195	92	103	68

Site #1: Legacy Research, Site #2: Trinity College, and Site #3: the University of Alabama at Birmingham.

### Functional-level similarity

To infer the significance of DNA methylation changes in the absence of definitive overlap, we next identified and compared overrepresented biological functions; the purpose was to identify pathway-level functional changes that may be related to experimental variables of interest. To assess the functional-level similarity between DMGs and DEGs identified as divergent between study sites, and attempt to determine if these represented true epigenetic changes associated with experimental variables, enrichment analysis was performed using a hypergeometric test with our in-house R analysis package *richR* (http://github.com/hurlab/richR) in terms of Gene Ontology (GO) terms^13,14^ and Kyoto Encyclopedia of Genes and Genomes (KEGG) pathways^15^. The complete lists of significant GO terms and KEGG pathways overrepresented in DMGs and DEGs are given in Supplementary Tables S14-S25. Supplementary Table S26 summarized the numbers of significant biological functions identified in each gene set. DMGs have over 400 significant GO terms with adjusted* p* < 0.05 but very few significant KEGG pathways.

**Figure 4:**
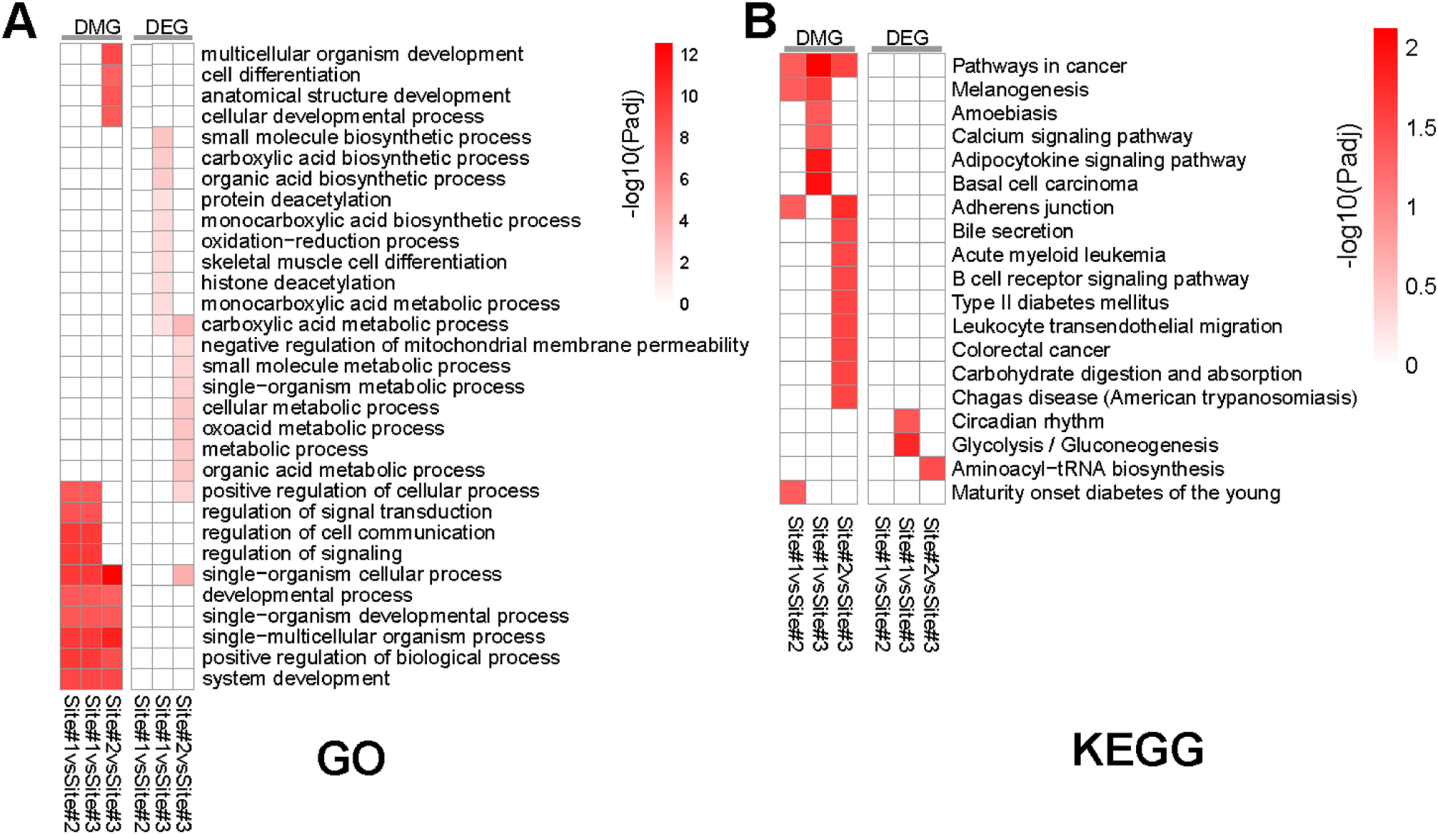
Enriched biological functions in terms of GO terms and KEGG pathways. Functional enrichment analysis was performed using *richR*, our in-house R package, on each of the DMG and DEG sets to identify over-represented biological functions in terms of GO terms (**A**) and KEGG pathways (**B**). Top 10 most significant GO terms and all significant KEGG pathways were combined in heatmaps, in which the color corresponds to enrichment significance represented by −log_10_(Benjamini–Hochberg (BH)-adjusted *P* values). Panel images were created using R (v4.0.3).

Heatmaps were generated with top significant functions, where colored cells indicated significant enrichment within the corresponding dataset (Fig. 4). The top enriched GO terms were very similar across all three DMG sets (Fig. 4A), while very few GO terms were enriched partially due to the small numbers of DEGs. KEGG analysis revealed different sets of pathways were enriched in these gene sets. “Pathways in cancer” was identified in all three DMG sets, but no apparent theme was identified.

## Discussion

Each of the four major categories of experimental/environmental factors used in the present study identified noticeable differences in the methylome and transcriptome. In the “before-each-laboratory” category, the animal vendors were different; Charles River Laboratory for Sites #1 and #2 and Harlan (Envigo) for Site #3. Although we purchased Sprague–Dawley rats from both vendors, there could be vendor-specific genetic differences. Studies have shown that many animal models of the same strains could have phenotypic as well as genetic variances according to the sources (vendors)^16–18^. In addition to possible genetic differences, these two vendors used different chows (Purina 5L79 at Charles River and Teklad Global 18% Protein Rodent Diet at Envigo). Although we did not examine specifically the possible impact of animal chow on the epigenome, other studies have reported differences in animal phenotypes resulting from changes in nutrition; DNA methylation was labile in response to nutritional influence^19,20^. Sites #1 and #2 used the same animal vendor as well as the same branded chow (LabDiet 5001) at their laboratories, while Site#3 used a different animal vendor and a different selection of chow (NIH open formula rat sterilizable diet). These differences are well aligned with the more outstanding differences in methylome and transcriptome between Site #3 and other sites.

Other noticeable factors include days-from-arrival-to-sacrifice in the “up-to-sacrifice” category and type-of-buffer and the use of air bubbles in the “dissection” category. Rats were killed and hippocampi dissected 27 days after arrival from the vendor in Site #3, while rats in Sites #1 and #2 were killed and hippocampi dissected 18 to 19 days after arrival. Aging is known to be correlated with epigenetic changes, particularly DNA methylation^21,22^. This difference may be associated with different body weights, which also showed significant differences across the project sites and correlated with different methylation levels of DMCs (Supplementary Fig. 3).

There is a possibility that the delay in sacrificing by 8 to 9 days along with changes in body weight affected the DNA methylome; however, it is not clear the degree to which the changes were attributable to delay. As for the different factors in the “dissection” category, Site #3 used artificial cerebrospinal fluid as their choice of a buffer solution with 95% O_2_ and 5% CO_2_ bubbled, while the other two sites used 0.9% saline. Although hyperoxia may result in genome-wide changes in DNA-methylation^23^, the effect of relatively short-term exposure during dissection on methylation change would not be expected to be substantial due to the time it generally takes to see methylation changes in culture.

Changes in methylation and gene expression can be either protocol-induced variations or could be considered epigenetic noise. Accordingly, we examined changes in pathways and biological functions using GO and KEGG analyses. No GO term changes related to DNA methylation were significantly enriched. However, one GO term that was changed was histone deacetylation; it was significantly enriched among the DEGs between Site #1 (Legacy) and Site #3 (UAB). Six DEGs, including *Per1*, *Per2*, *Rbm14*, *Bcl6*, *Sfpq*, and *Prkd2,* were included in this set, suggesting a potential difference in another epigenetic marker of histone deacetylation is taking place.

Nearly identical protocols (Sites #1 and #2) resulted in a close match of DNA methylation and RNA-seq profiles, whereas seemingly minor differences induced major changes in the DNA methylome and transcriptome. Although it is impossible to gauge the extent of their individual or combined effects on DNA methylation changes in this study, some of these factors have been implicated in modulating epigenetic changes^20^.

We also examined the relationship between the significant changes in DNA methylation and significant expression change in the nearest gene, based on the assumed inverse correlation between methylation and gene expression. Little overlap was observed up to 5% of DMGs with DEGs, approximately 59% of them showed an inverse relationship (increased methylation with down-regulated gene expression or decreased methylation with up-regulated gene expression), suggesting that the directionality of gene expression change cannot and should not be inferred based solely on whether DNA methylation is increased or decreased at a particular site. Associating DNA methylation to gene expression is very challenging and DNA methylation often has a strong influence through most distal effects as at enhancer elements or CTCF binding sites^24,25^; therefore, our current overlap analysis has room for improvement.

Bioinformatics approaches for addressing the batch differences in certain high-throughput data analysis are available by using either tools such as ComBat^26^, SVA^27^, and removeBatchEffect^28^, or including the batch information as a covariate. However, caution is needed when using batch correction methods as they may bias the data in unpredicted ways^29^. Our study demonstrates that seemingly minor experimental variations, even under normal baseline conditions, can have a significant impact on epigenome outcome measures and data interpretation. This is particularly meaningful for neurological studies in animal models, in which baseline parameters between experimental groups are difficult to control^10^. Therefore, strict guidelines for the execution and interpretation of epigenetic studies, possibly including additional controls in experimental design to adjust protocol-sensitive epigenetic, are needed to enhance scientific rigor, and these data identify protocol-sensitive epigenetic changes that may confound experimental results.

## Methods

### Animals

SAS Sprague Dawley (SD) male rats were purchased from the nearest vendors (Charles River and Envigo) from our three project sites, including Legacy Research (Site #1), Trinity College (Site #2), and the University of Alabama at Birmingham (Site #3). Animal handling was approved by the Institutional Animal Care and Use Committee (IACUC) at each of three sites and summarized as listed in Table 1. The investigation conformed to the National Research Council of the National Academies Guide for the Care and Use of Laboratory Animals^30^ and complied with the ARRIVE guidelines.

### RRBS and RNA-Seq

Whole hippocampi from each animal were surgically dissected and flash-frozen in liquid N_2_ and stored at -80 °C before being shipped to the University of North Dakota (UND), where samples were collected, de-identified, and stored at -80 °C for deep sequencing analysis. Once collected, all samples were processed using Qiagen’s AllPrep DNA/RNA Mini Kit (Germantown, MD; Product ID: 80,204) to individually extract RNA and DNA from each flash-frozen sample. All RNA and DNA samples were stored at -80 °C before being sent frozen in dry ice to the University of Michigan Genomics and Epigenomics Core for deep sequencing.

The RRBS procedure was adapted as previously described and performed at the University of Michigan Epigenetics Core facility^31^. Briefly, genomic DNA quantity was measured using a Qubit fluorometer (ThermoFisher Scientific, Waltham, MA) and the quality assessed using TapeStation (Agilent, Santa Clara, CA). Genomic DNA was digested with *Msp1* restriction enzyme and purified using phenol:chloroform extraction and ethanol precipitation. Following *Msp1* digestion, genomic DNA underwent blunt-end digestion, phosphorylation, and ligation of adapters with methylated cytosines. Ligated fragments, processed for size selection using agarose gel electrophoresis, were bisulfite converted, amplified by PCR, then cleaned using AMPure XP beads (Beckman Coulter Life Sciences, Indianapolis, IN). Libraries were quantified using Qubit fluorometric quantification (ThermoFisher Scientific, Waltham, MA), analyzed using a TapeStation system (Agilent, Santa Clara, CA), then sequenced on an Illumina Hi-Seq 4000 platform (Illumina, San Diego, CA).

Total RNA isolated from individual microglia preparations was evaluated using a TapeStation system (Agilent, Santa Clara, CA). The NEBNext Ultra II RNA Library Prep Kit for Illumina (New England Biolabs, Ipswich, MA) was used to construct the sequencing library. Resultant cDNA was commercially sequenced with a paired-end read length of 50 bases using an Illumina Hi-Seq 4000 platform (Illumina, San Diego, CA).

### RRBS and RNA-Seq data processing

Quality control assessment of RRBS data was completed using *FastQC* v0.11.5^32^. Raw Sequencing reads were cleaned using *Trim Galore* v0.5.0, to remove reads with adapter contamination and reads with a Phred quality score less than 30^33^. Cleaned reads were mapped to an in silico bisulfite-converted rat reference genome rn6 using *Bismark* v0.20.0 and *Bowtie2* v2.3.4.2^34,35^. CpG sites on X and Y chromosomes were excluded. PCA was performed on the most variant 10% of the measured CpG sites. *methylKit* v1.8.1^36^ was used to count uniquely mapped reads and assess changes in methylation between each site. Differentially methylated CpGs (DMCs) were defined as a 25% or greater difference in cytosine methylation levels between sites with an adjusted *p* value < 0.01, which were then aggregated into differentially methylated genes (DMGs) based on the unique gene identifiers. DMCs were annotated based on genes and CpG island features using gene bodies and 2, 5, and 10 kb regions upstream from transcription start sites using the *genomation* R package^37^. Annotation of murine CpG islands was obtained from the University of California, Santa Cruz, CA (UCSC, https://genome.ucsc.edu/cgi-bin/hgGateway?db=rn6). The gene annotation was obtained from Ensembl and NCBI gene databases^38^.

Quality control assessment of RNA-Seq data was completed using the *FastQC* v0.11.5^32^. Raw sequencing reads were cleaned using *Trimmomatic*^39^ to remove reads with adapters or poly-N sequences as well as reads with quality scores < 30. Cleaned reads were mapped to the rat reference genome rn6 using *HISAT2*^40^. Genes with zero expression across samples were omitted from differential expression analysis. featureCounts was used to assign mapped reads to unique genomic features^41^. PCA was performed to gain insights into the association between samples. Differentially expressed genes (DEGs) were identified using the *DESeq2* R package with a significance cutoff of < 0.05 adjusted *p* value^42^.

### Hippocampus region-specific expression markers

To assess the possibility of imbalance in the dissected hippocampal regions, which might have resulted in the observed methylation and expression differences, we examined the expression levels of region-specific hippocampal markers. We compiled 187 region-specific markers from the Hipposeq, a comprehensive RNA-Seq database of gene expression in hippocampal principal neurons^11^. This list includes 10 CA1-enriched, 41 CA2-enriched, 61 CA4 enriched, 39 dentate gyrus-enriched, 12 dorsal-enriched, and 24 ventral-enriched genes (Supplementary Table S2). A heatmap of expression levels of these marker genes was created based on the Fragments Per Kilobase Million (FPKM) values.

### Cell-type composition analysis

To assess the cell-type composition difference of the samples across three sites, the expression levels of selected known neuronal cell-type-specific markers for microglia, astrocytes, neurons, oligodendrocytes, as well as those are known to be expressed in various cell types. These marker genes were compiled in our recent study^43^ on alpha-synuclein-associated differential methylation signatures in microglia, which employed two high-throughput expression profiling studies in rodent brains^44,45^. We also employed CIBERSORT^12^ to infer the cell type abundance based on gene expression- and marker genes. Brain cell-type-specific signatures of 903 genes were obtained from a study in human brains^46^, which included astrocytes, endothelial, fetal quiescent, fetal replicating, microglia, neurons, oligodendrocytes, oligodendrocyte progenitor cells (OPC). These human gene signatures were mapped to rat genes based on the Ensembl Genes database v104 annotation using the *biomaRt* Bioconductor package^47^. CIBERSOFT function available in *IOBR* R package was used to estimate the abundances of the member cell type from the RNA-Seq data. Kruskal–Wallis test was used to examine the significant difference in the cell-type composition across the samples.

### Functional enrichment analysis

To identify significantly over-represented biological functions, a functional enrichment analysis of both DMGs and DEGs was conducted using our in-house enrichment analysis R package *richR* (http://github.com/hurlab/richR). Gene Ontology (GO)^48^ and Kyoto Encyclopedia of Genes and Genomes (KEGG)^49^ were used as the target annotation sources of biological functions and pathways. A Benjamini–Hochberg adjusted *p* value of < 0.05 was used as the significance cutoff. *VennDetail* Bioconductor package^50^ was used to examine the overlap at the biological functions/pathways as well as at the gene level (DEGs and DMGs).

## Supplementary Information

Supplementary Information 1.

Supplementary Information 2.

## Data Availability

The raw sequencing data have been deposited into the NCBI Gene Expression Omnibus database (Accession ID: GSE164833). Analysis scripts used in the current study are freely available at our GitHub site (https://github.com/hurlab/ProtocolMatters). All other data generated or analyzed during this study are included in this article and its supplementary materials.

## References

[1] Garber K (2019). Epigenetics comes to RNA. Science.

[2] Qureshi IA, Mehler MF (2018). Epigenetic mechanisms underlying nervous system diseases. Handb Clin Neurol.

[3] Williams-Karnesky RL (2013). Epigenetic changes induced by adenosine augmentation therapy prevent epileptogenesis. J. Clin. Invest..

[4] Robinson GE, Barron AB (2017). Epigenetics and the evolution of instincts. Science.

[5] O'Reilly S (2019). Epigenetic modulation as a therapy in systemic sclerosis. Rheumatology (Oxford).

[6] Younus I, Reddy DS (2017). Epigenetic interventions for epileptogenesis: A new frontier for curing epilepsy. Pharmacol. Ther..

[7] Ahuja N, Sharma AR, Baylin SB (2016). Epigenetic therapeutics: A new weapon in the war against cancer. Annu. Rev. Med..

[8] Rezapour S, Hosseinzadeh E, Marofi F, Hassanzadeh A (2019). Epigenetic-based therapy for colorectal cancer: Prospect and involved mechanisms. J. Cell Physiol..

[9] Zahnow CA (2016). Inhibitors of DNA methylation, histone deacetylation, and histone demethylation: A perfect combination for cancer therapy. Adv. Cancer Res..

[10] Debski KJ (2016). Etiology matters—Genomic DNA methylation patterns in three rat models of acquired epilepsy. Sci. Rep..

[11] Cembrowski MS, Wang L, Sugino K, Shields BC, Spruston N (2016). Hipposeq: A comprehensive RNA-seq database of gene expression in hippocampal principal neurons. Elife.

[12] Newman AM (2019). Determining cell type abundance and expression from bulk tissues with digital cytometry. Nat. Biotechnol..

[13] Ashburner M (2000). Gene ontology: Tool for the unification of biology. The Gene Ontology Consortium. Nat. Genet.

[14] Gene Ontology C (2021). The Gene Ontology resource: Enriching a GOld mine. Nucleic Acids Res..

[15] Kanehisa M, Goto S (2000). KEGG: Kyoto encyclopedia of genes and genomes. Nucleic Acids Res..

[16] Zhang-James Y, Middleton FA, Faraone SV (2013). Genetic architecture of Wistar-Kyoto rat and spontaneously hypertensive rat substrains from different sources. Physiol. Genom..

[17] Kiselycznyk C, Holmes A (2011). All (C57BL/6) mice are not created equal. Front. Neurosci..

[18] Oliff HS, Coyle P, Weber E (1997). Rat strain and vendor differences in collateral anastomoses. J. Cereb. Blood Flow Metab..

[19] Kadayifci FZ, Zheng S, Pan YX (2018). Molecular mechanisms underlying the link between diet and DNA methylation. Int. J. Mol. Sci..

[20] Zhang N (2015). Epigenetic modulation of DNA methylation by nutrition and its mechanisms in animals. Anim. Nutr..

[21] Unnikrishnan A (2019). The role of DNA methylation in epigenetics of aging. Pharmacol. Ther..

[22] Lowe R (2020). DNA methylation clocks as a predictor for ageing and age estimation in naked mole-rats, Heterocephalus glaber. Aging (Albany NY).

[23] Chen CM, Liu YC, Chen YJ, Chou HC (2017). Genome-wide analysis of DNA methylation in hyperoxia-exposed newborn rat lung. Lung.

[24] Ordonez R, Martinez-Calle N, Agirre X, Prosper F (2019). DNA methylation of enhancer elements in myeloid neoplasms: Think outside the promoters?. Cancers (Basel).

[25] Heberle E, Bardet AF (2019). Sensitivity of transcription factors to DNA methylation. Essays Biochem..

[26] Johnson WE, Li C, Rabinovic A (2007). Adjusting batch effects in microarray expression data using empirical Bayes methods. Biostatistics.

[27] Leek JT, Johnson WE, Parker HS, Jaffe AE, Storey JD (2012). The sva package for removing batch effects and other unwanted variation in high-throughput experiments. Bioinformatics.

[28] Ritchie ME (2015). limma powers differential expression analyses for RNA-sequencing and microarray studies. Nucleic Acids Res..

[29] Nygaard V, Rodland EA, Hovig E (2016). Methods that remove batch effects while retaining group differences may lead to exaggerated confidence in downstream analyses. Biostatistics.

[30] Council, N. R. *Guide for the Care and Use of Laboratory Animals*. (National Academies Press, 2010).

[31] Garrett-Bakelman FE (2015). Enhanced reduced representation bisulfite sequencing for assessment of DNA methylation at base pair resolution. J Vis Exp..

[32] Bioinformatics, B. *FastQC: A quality control tool for high throughput sequence data.* http://www.bioinformatics.babraham.ac.uk/projects/fastqc/.

[33] Bioinformatics, B. *Trim Galore!* https://www.bioinformatics.babraham.ac.uk/projects/trim_galore/

[34] Krueger F, Andrews SR (2011). Bismark: A flexible aligner and methylation caller for Bisulfite-Seq applications. Bioinformatics (Oxford, England).

[35] Langmead B, Salzberg SL (2012). Fast gapped-read alignment with Bowtie 2. Nat. Methods.

[36] Akalin A (2012). methylKit: A comprehensive R package for the analysis of genome-wide DNA methylation profiles. Genome Biol..

[37] Akalin A, Franke V, Vlahovicek K, Mason CE, Schubeler D (2015). Genomation: A toolkit to summarize, annotate and visualize genomic intervals. Bioinformatics.

[38] Zerbino DR (2018). Ensembl 2018. Nucleic Acids Res..

[39] Bolger AM, Lohse M, Usadel B (2014). Trimmomatic: A flexible trimmer for Illumina sequence data. Bioinformatics.

[40] Kim D, Langmead B, Salzberg SL (2015). HISAT: A fast spliced aligner with low memory requirements. Nat. Methods.

[41] Liao Y, Smyth GK, Shi W (2014). featureCounts: An efficient general purpose program for assigning sequence reads to genomic features. Bioinformatics.

[42] Love MI, Huber W, Anders S (2014). Moderated estimation of fold change and dispersion for RNA-seq data with DESeq2. Genome Biol..

[43] McGregor BA (2021). Alpha-synuclein-induced DNA methylation and gene expression in microglia. Neuroscience.

[44] Ximerakis M (2019). Single-cell transcriptomic profiling of the aging mouse brain. Nat. Neurosci..

[45] Zhang Y (2014). An RNA-sequencing transcriptome and splicing database of glia, neurons, and vascular cells of the cerebral cortex. J. Neurosci..

[46] Yu Q, He Z (2017). Comprehensive investigation of temporal and autism-associated cell type composition-dependent and independent gene expression changes in human brains. Sci. Rep..

[47] Durinck S (2005). BioMart and Bioconductor: A powerful link between biological databases and microarray data analysis. Bioinformatics.

[48] Ashburner M (2000). Gene ontology: Tool for the unification of biology. The Gene Ontology Consortium. Nat. Genet..

[49] Kanehisa M, Furumichi M, Tanabe M, Sato Y, Morishima K (2017). KEGG: New perspectives on genomes, pathways, diseases and drugs. Nucleic Acids Res..

[50] Guo, A., McGregor, B. A. & Hur, J. *VennDetail: A package for visualization and extract details.* https://www.bioconductor.org/packages/release/bioc/html/VennDetail.html (2021).

