# Supplementary material for: The impact of methodology on the reproducibility and rigor of DNA methylation data
Source: Sci Rep. 2022 Jan 10;12:380. doi: 10.1038/s41598-021-04346-w (PMC8748700; doi:10.1038/s41598-021-04346-w)
Supplement: Supplementary file 2 — Supplementary Information 2. [file 41598_2021_4346_MOESM2_ESM.docx]

Supplementary Materials for

**The impact of methodology on the reproducibility and rigor of DNA methylation data**

Detlev Boison*, Susan A. Masino*, Farah D. Lubin*, Kai Guo*, Theresa Lusardi, Richard Sanchez, David N. Ruskin, Joyce Ohm, Jonathan D. Geiger, and Junguk Hur^$^

Correspondence to: [junguk.hur@med.und.edu](mailto:junguk.hur@med.und.edu)

**This PDF file includes:**

Supplementary Fig. 1

Supplementary Fig. 2

Supplementary Fig. 3

Supplementary Fig. 4

**Other Supplementary Materials for this manuscript include the following:**

Supplementary Tables 1 to 26 (Assembled in a single Excel file)


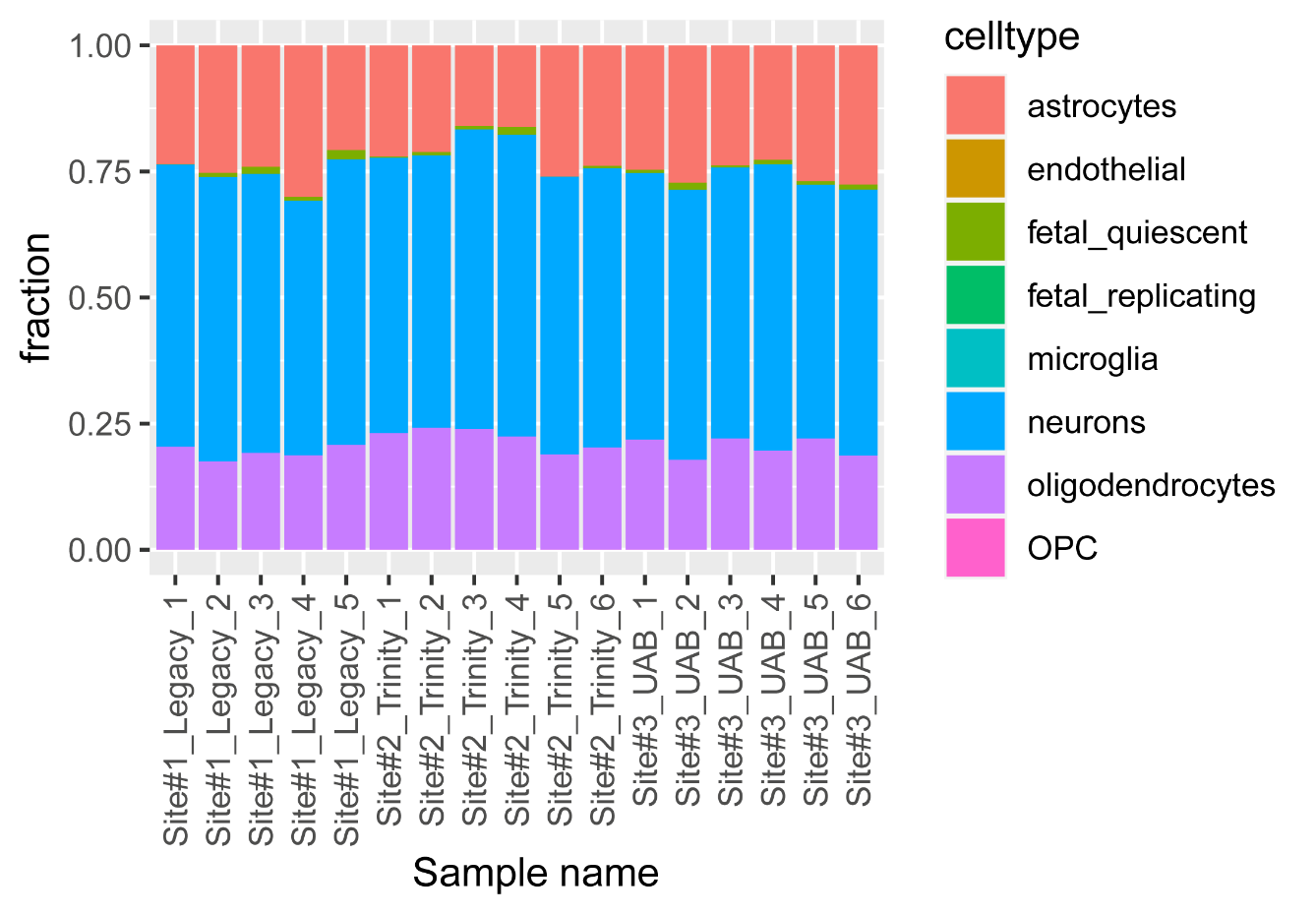


**Supplementary Fig. 1. Cell-type abundance based on the expression data**. To assess the cell-type composition of the hippocampal tissues used in the study, we employed a gene expression- and marker gene-based approach using the CIBERSORT algorithm. The brain cell-type-specific 903 gene expression signatures for CIBERSORT were obtained from a study in human brains, including eight cell types of astrocytes, endothelial, fetal quiescent, fetal replicating, microglia, neurons, oligodendrocytes, oligodendrocyte progenitor cells (OPC). CIBERSOFT function available in IOBR R package was used to estimate the abundances of the member cell type from the RNA-Seq data.

**
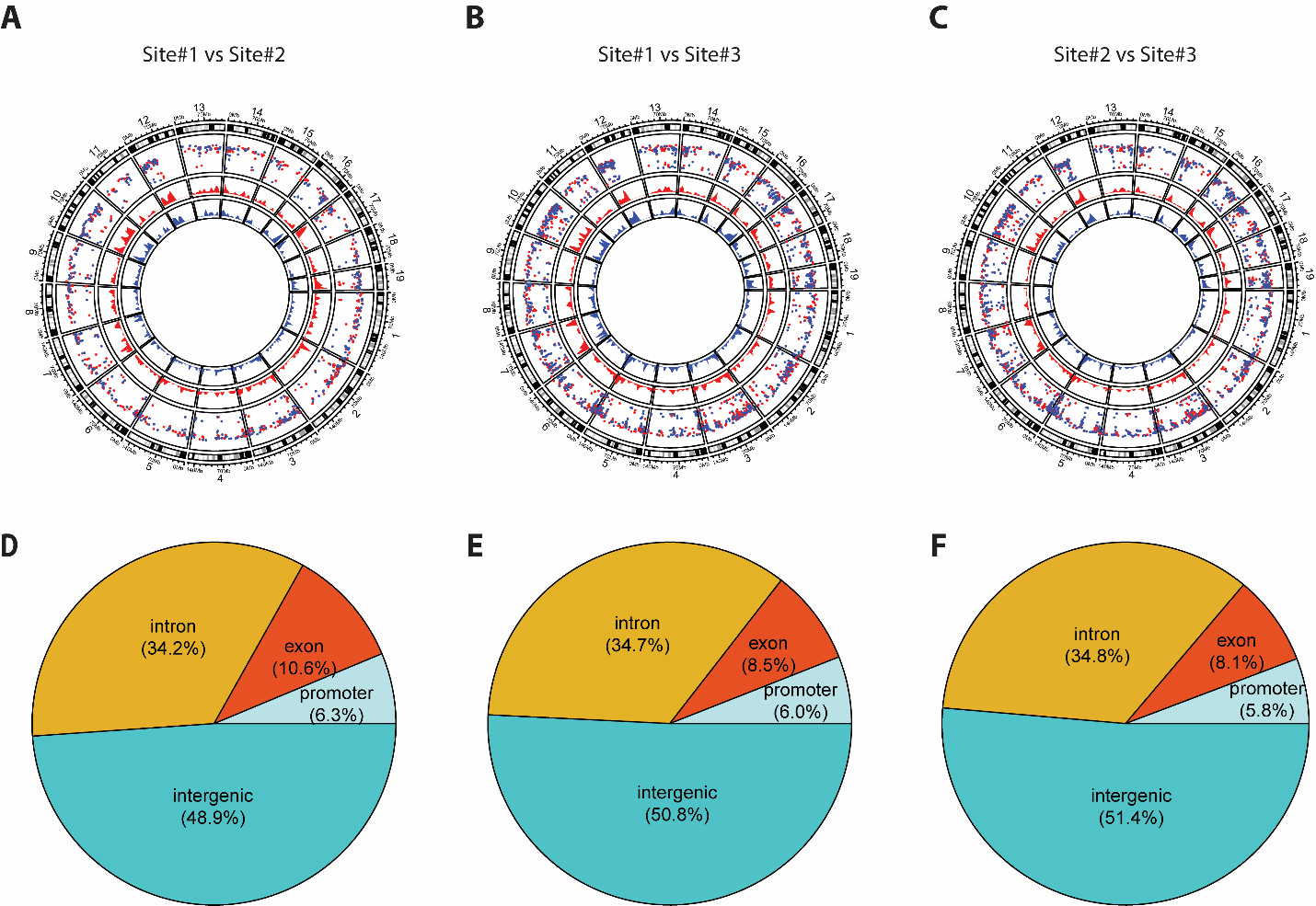
**

**Supplementary Fig. 2. Distribution of differentially methylated CpGs (DMCs)**. DMCs (A: Site #1 vs Site #2; B: Site #1 vs Site #3; C: Site #2 vs Site #3) across chromosomes are depicted in circular plots. Hyper- and hypomethylated CpGs are colored in red and blue, respectively, relative to their second Sites in each comparison. The distributions of DMCs are summarized based on genomic location (D-F). These plots were generated using R v4 (<https://cran.r-project.org/>).


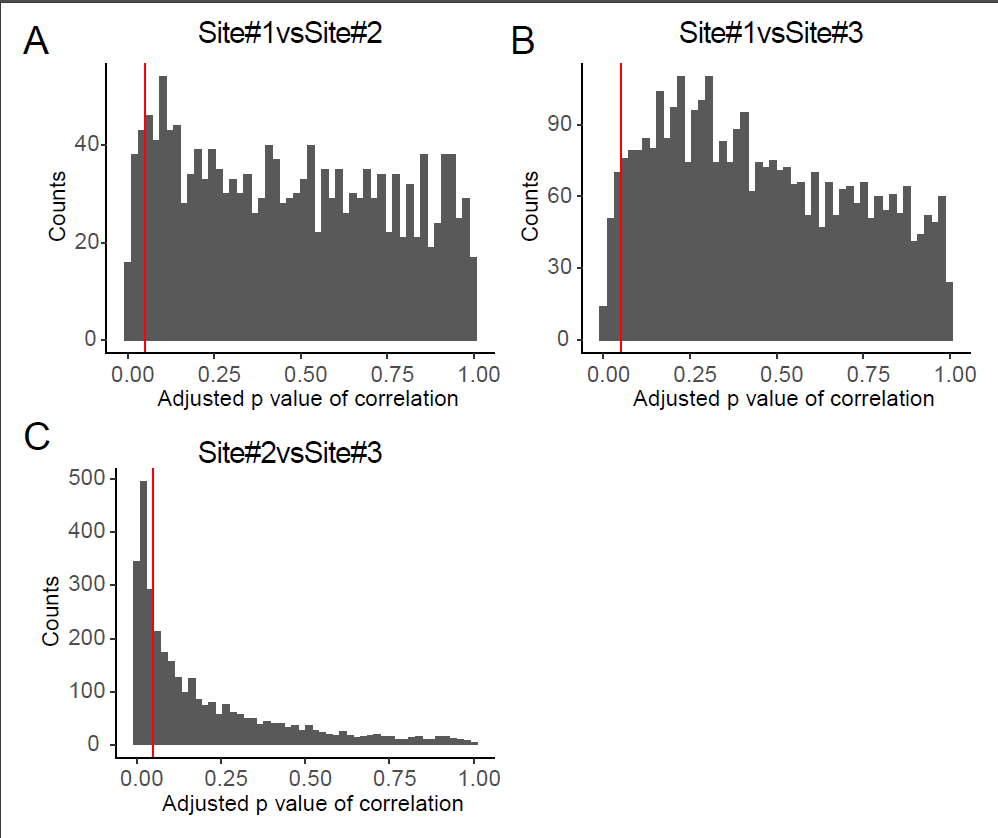


**Supplementary Fig. 3. Distribution of correlation between the methylation level of DMCs and body weight**. Pearson correlation analysis was done between the methylation level of DMCs and body weight of animals right before being sacrificed was the distribution of adjusted p-values of these correlations are depicted in histograms (A: Site #1 vs Site #2; B: Site #1 vs Site #3; C: Site #2 vs Site #3). The vertical red lines indicate the adjusted p-value = 0.05. While the majority of the correlations were not significant, a larger portion in the Site#2 vs Site#3 comparison (C) was significant (adjusted P < 0.05). These plots were generated using R v4 (<https://cran.r-project.org/>).

**
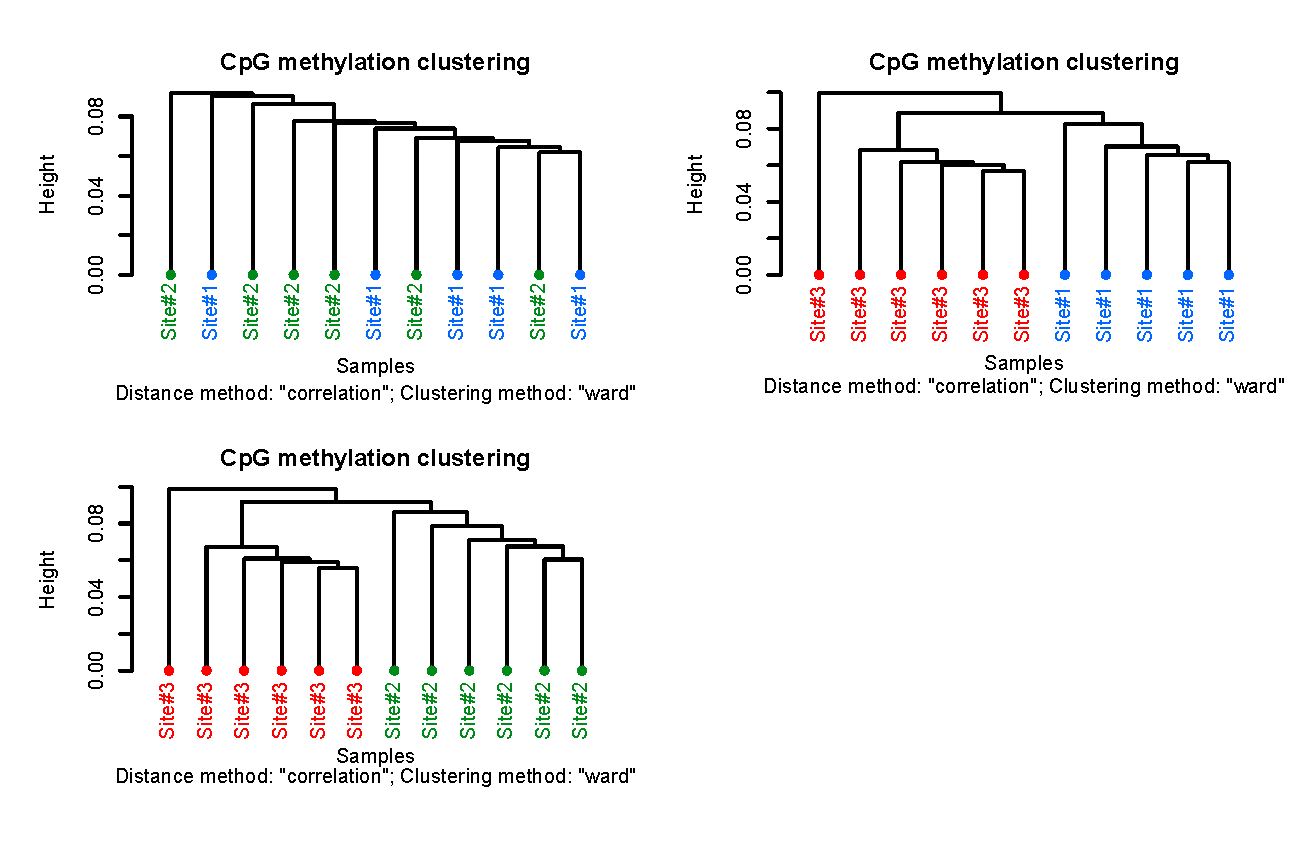
**

**Supplementary Fig. 4. Hierarchical clustering of CpG methylation data**. These clustering results suggest the high similarity between the samples from Site#1 (Legacy Institute) and Site#2 (Trinity College), but a separation of the sites from Site#3 (University of Alabama at Birmingham). These plots were generated using R v4 (<https://cran.r-project.org/>).

**List of Supplementary Tables (single Excel file)**

**Supplementary Table 1:** CpG site mapping summary

**Supplementary Table 2:** Region-specific hippocampus markers

**Supplementary Table 3.** Average expression of neuronal cell-type-specific marker genes

**Supplementary Table 4.** Assessment of differential cell-type abundance across three project sites

**Supplementary Table 5:** Differentially methylated genes between Site #1 (Legacy) and Site #2 (Trinity)

**Supplementary Table 6:** Differentially methylated genes between Site #1 (Legacy) and Site #3 (UAB)

**Supplementary Table 7:** Differentially methylated genes between Site #2 (Trinity) and Site #3 (UAB)

**Supplementary Table 8:** Differentially expressed genes between Site #1 (Legacy) and Site #2 (Trinity)

**Supplementary Table 9:** Differentially expressed genes between Site #1 (Legacy) and Site #3 (UAB)

**Supplementary Table 10:** Differentially expressed genes between Site #2 (Trinity) and Site #3 (UAB)

**Supplementary Table 11:** Differentially methylated regions between Site #1 (Legacy) and Site #2 (Trinity)

**Supplementary Table 12:** Differentially methylated regions between Site #1 (Legacy) and Site #3 (UAB)

**Supplementary Table 13:** Differentially methylated regions between Site #2 (Trinity) and Site #3 (UAB)

**Supplementary Table 14:** Gene ontology enrichment result of DMGs between Site #1 (Legacy) and Site #2 (Trinity)

**Supplementary Table 15:** Gene ontology enrichment result of DMGs between Site #1 (Legacy) and Site #3 (UAB)

**Supplementary Table 16:** Gene ontology enrichment result of DMGs between Site #2 (Trinity) and Site #3 (UAB)

**Supplementary Table 17:** Gene ontology enrichment result of DEGs between Site #1 (Legacy) and Site #2 (Trinity)

**Supplementary Table 18:** Gene ontology enrichment result of DEGs between Site #1 (Legacy) and Site #3 (UAB)

**Supplementary Table 19:** Gene ontology enrichment result of DEGs between Site #2 (Trinity) and Site #3 (UAB)

**Supplementary Table 20:** KEGG pathway enrichment result of DMGs between Site #1 (Legacy) and Site #2 (Trinity)

**Supplementary Table 21:** KEGG pathway enrichment result of DMGs between Site #1 (Legacy) and Site #3 (UAB)

**Supplementary Table 22:** KEGG pathway enrichment result of DMGs between Site #2 (Trinity) and Site #3 (UAB)

**Supplementary Table 23:** KEGG pathway enrichment result of DEGs between Site #1 (Legacy) and Site #2 (Trinity)

**Supplementary Table 24:** KEGG pathway enrichment result of DEGs between Site #1 (Legacy) and Site #3 (UAB)

**Supplementary Table 25:** KEGG pathway enrichment result of DEGs between Site #2 (Trinity) and Site #3 (UAB)

**Supplementary Table 26:** GO and KEGG enrichment summary statistics
